# Knowledge, attitutes and perceptions of medical and pharmacy students about HIV pre-exposure prophylaxis: a cross-sectional survey from Pakistan

**DOI:** 10.1186/s12909-025-08338-6

**Published:** 2025-11-29

**Authors:** Usama Idrees, Muhammad Wasay Shahid, Faizur Rehman, Zaid Ahmad, Bilal Ahmad, Aysha Iftikhar, Talha Ashraf Zia, Humza Saeed, Arslan Ahmed, Tayyaba Shahbaz, Ali Ahmed

**Affiliations:** 1 Khawaja Muhammad Safdar Medical College, Sialkot, Punjab Pakistan; 2 Riphah Institute of Pharmaceutical Sciences, Riphah International University, Islamabad, Pakistan; 3 Saidu Medical College, Saidu Sharif, Swat District, Khyber Pakhtunkhwa, Pakistan; 4 Faisalabad Medical University, Faisalabad, Punjab Pakistan; 5 Southeast Georgia Health System, Brunswick, GA USA; 6 King Edward Medical College, Lahore, Punjab Pakistan; 7 Rawalpindi Medical University, Rawalpindi, Punjab Pakistan; 8 Division of Infectious Diseases and Global Public Health, School of Medicine, University of California San Diego (UCSD), San Diego, USA

**Keywords:** Pre-exposure prophylaxis, PrEP, HIV prevention, Knowledge, Attitude, And perceptions, KAP, Medical students, Pharmacy students, Pakistan

## Abstract

**Background:**

Despite the rising HIV burden in Pakistan, utilization of HIV pre-exposure prophylaxis (PrEP) remains critically low. As future healthcare providers, medical and pharmacy students play a pivotal role in expanding PrEP access. Yet, their readiness to integrate PrEP into practice remains underexplored. This study assessed knowledge, attitudes, and perceptions (KAP) of clinical-year medical and pharmacy students regarding PrEP in Pakistan.

**Methods:**

A cross-sectional survey was conducted between May and June 2025 among fourth- and final-year medical and pharmacy students across Pakistan. A validated, structured questionnaire evaluated PrEP-related knowledge, attitudes, and perceptions. Descriptive statistics summarized participant characteristics, Mann-Whitney U tests, and Spearman’s correlation examined associations between demographic factors and KAP scores.

**Results:**

A total of 359 responses were analyzed; 70.5% reported PrEP awareness. The mean knowledge score was modest (6.54/10), with over 50% mistakenly identifying PrEP as a vaccine and 55.6% believing HIV testing is unnecessary before initiation. Medical students demonstrated significantly higher in knowledge (*p* < 0.001), while pharmacy students exhibited more favorable attitudes (*p* < 0.001) and perceptions (*p* = 0.023). A strong positive correlation was observed between attitudes and perceptions (*r* = 0.555, *p* < 0.001), but knowledge did not significantly correlate with either. Encouragingly, most participants supported PrEP promotion (87.4%), government-funded provision (84.7%), and inclusion in HIV education (82.7%).

**Conclusion:**

While both medical and pharmacy students expressed supportive attitudes and accurate perceptions of PrEP, considerable knowledge gaps persist, particularly among pharmacy students, posing barriers to effective PrEP implementation. Misconceptions regarding PrEP’s mechanism and initiation protocols highlight curricular shortcomings, especially in pharmacy education. Integrating evidence-based, stigma-sensitive, and case-based HIV prevention modules into health professional curricula is critical. Strengthening training at the undergraduate level will better equip future healthcare providers to champion PrEP delivery, thereby enhancing PrEP uptake and HIV prevention in Pakistan.

**Supplementary Information:**

The online version contains supplementary material available at 10.1186/s12909-025-08338-6.

## Background

Pre-exposure Prophylaxis (PrEP) has emerged as a pivotal advancement in the global strategy to prevent HIV infection, particularly among individuals at high risk. Since its introduction and regulatory approval, PrEP has transformed HIV prevention through daily administration of antiretroviral medications, typically a combination of tenofovir and emtricitabine [1]. By blocking HIV replication at an early stage, PrEP significantly reduced the risk of seroconversion [2]. Multiple clinical trials and real-world studies have consistently demonstrated its effectiveness in preventing HIV transmission among key populations (KPs), including men who have sex with men (MSM), people who inject drugs (PWID), transgender individuals, and serodiscordant couples [3]. Efficacy rates of 90% to 92% have been documented with high adherence levels, affirming PrEP as a cornerstone intervention in HIV prevention [4].

In recognition of PrEP’s efficacy, the World Health Organization (WHO) and Centers for Disease Control and Prevention (CDC) have issued comprehensive guidelines promoting its integration into national HIV prevention programs. The guidelines recommend tenofovir-based oral PrEP formulations, administered daily, along with supportive strategies such as adherence counselling and harm reduction services [3, 5]. WHO has updated its provider module to reinforce the use of oral PrEP and expand clinical guidance to two additional modalities, the dapivirine vaginal ring (DVR) and long-acting cabotegravir (CAB-LA), offering broader options for diverse key populations and healthcare settings [6]. Although 94% of WHO member states have adopted PrEP policy, actual program coverage remains uneven [7]. Disparities in implementation are influenced by a range of structural, social, and healthcare system-related barriers [8]. In particular, health system constraints and provider-related challenges continue to limit PrEP delivery and scale-up, especially in low-or middle-income countries (LMICs) [9].

Although awareness has been increased among healthcare providers, including infectious disease specialists, primary care physicians, and internal medicine practitioners, this trend has not been matched by a proportional increase in prescribing behaviors [10]. Research has consistently highlighted that limited knowledge, suboptimal training, and persistent stigma among healthcare professionals are significant barriers to effective PrEP provision [11]. A study in 2020 revealed that 40% of individuals who were denied access to PrEP experienced such outcomes due to the clinician’s lack of appropriate knowledge and training [12]. Among medical students, a notable proportion, approximately one-third, harbor stigmatizing attitudes towards people with HIV (PWH), which has been strongly correlated with insufficient curricular content and outdated instructional methods in medical education [13]. These deficiencies not only hinder effective patient care but also perpetuate negative stereotypes, further limiting the reach and acceptance of PrEP.

At the same time, there is evidence of progress within medical practice. Programs have successfully embedded PrEP services within existing sexual health workflows, leading to increased prescribing rates. For instance, a study noted a rise in PrEP prescriptions from 12 to 51 among providers after implementing a structured program [14]. Studies have shown that training programs and clinical support decision tools for healthcare providers have been linked to higher rates of PrEP prescriptions. Providers who received targeted training reported greater comfort and competence in prescribing PrEP, particularly among Black cisgender women, a population with historically low uptake [15]. Clinical support decision tools have been shown to facilitate PrEP delivery by improving providers’ ability to assess patient risk and make informed choices [16]. These tools can streamline the prescribing process, making it more efficient. Innovative service delivery models, such as telemedicine follow-ups [17] and integration into reproductive and maternal health services [18] have demonstrated that medical practice can be successfully adapted to normalize PrEP as a part of comprehensive preventive care.

The scope of pharmacy practice has expanded considerably in recent years, positioning pharmacists as potential facilitators of PrEP initiation and delivery [19]. However, many pharmacists report insufficient knowledge [20], low confidence [21], and limited experience [20], thus expressing the need for specialized training in identifying at-risk populations and managing PrEP-related care [22]. A 2018 study in the United States indicated that only 48% of pharmacists were aware of PrEP and just 33% had ever dispensed it [23]. The coronavirus-19 (COVID-19) pandemic has further accelerated the shift towards decentralized, community-based PrEP services, including pharmacy-led models. Although these models show promise, their successful implementation depends upon robust research evidence and targeted educational efforts [3].

In Pakistan, the HIV epidemic continues to escalate at an alarming pace. The country faces immense challenges in controlling the spread of the virus with an estimated 0.33 million individuals living with HIV and only around 55,652 receiving antiretroviral therapy (ART) [24]. In 2022, the national PrEP program was launched in collaboration with the United Nations; however, its progress has been impeded by societal stigma, limited health literacy, and widespread reluctance to engage with HIV care services, particularly those offered through ART centers [25]. Marginalized groups, including MSM, transgender individuals, and PWID, remain disproportionately affected by HIV, making it imperative to develop targeted prevention strategies [26].

Despite the escalating HIV burden, there is limited empirical research in Pakistan on the knowledge, attitudes, and perceptions (KAP) of healthcare professionals and students regarding PrEP. Curricular gaps in both medicine and pharmacy further constrain preparedness for HIV prevention. Assessing KAP among clinical-phase students is therefore essential for informing curricular reforms and capacity-building initiatives that strengthen the future healthcare workforce’s role in PrEP implementation.

## Methods

### Study design & study setting

This questionnaire-based study utilized a cross-sectional design to evaluate KAP of HIV PrEP among fourth and final year medical and pharmacy (PharmD). Participants were selected through convenience sampling from medical and pharmaceutical teaching institutes located in Islamabad and Faisalabad. The data was collected through convenience sampling from May to June 2025. To conduct this study, the Strengthening the Reporting of Observational Studies in Epidemiology (STROBE) checklist was followed and can be found in Additional File 1.

### Participants’ eligibility criteria

The study included undergraduate medical and pharmacy students enrolled in the fourth or final year of their respective degree programs at selected teaching institutions, provided they voluntarily gave informed consent and completed all sections of the questionnaire. Students from first to third year, those enrolled in non-medical or non-pharmacy programs, and respondents who submitted incomplete or duplicate responses or declined to participate were excluded from the study.

### Sample size estimation

The sample size was estimated using the Raosoft Online Calculator. Assuming a target population of 5000, a 5% margin of error, and a 95% confidence interval, the final number of participants came out to be 357. The population estimate was based on the approximate number of medical and pharmacy students enrolled across selected institutes. To account for the potential 10% non-response rate, the sample size was inflated to 397 using the formula:

$$\:N\left(adjusted\right)=\frac{N\:\left(unadjusted\:sample\:size\right)}{1-non\:response\:}=\frac{357}{1-0.10}=396.6\approx\:397$$

### Questionnaire design

A comprehensive, structured questionnaire was developed and uploaded to Google Forms (Google, LLC, Mountain View, California, United States) for online surveys. The questions were based on a thorough review of existing KAP literature and relevant HIV PrEP guidelines issued by the National AIDS Control Program, Pakistan (NACP) [27–31].

To ensure content validity, the questionnaire was reviewed by faculty members and researchers with expertise in infectious diseases, public health, and pharmacy practice. A pilot study was conducted among 30 medical and pharmacy students with equal proportions to assess clarity, relevance, and comprehension. Based on participant feedback, minor revisions were made to improve the wording and structure of the items. The internal consistency of the sub-scales was evaluated using Cronbach’s alpha, yielding values of 0.72 for the knowledge section, 0.71 for the attitude section, and 0.781 for the perception section, indicating acceptable reliability.

The final version of the questionnaire comprised twenty-nine items and was divided into the following segments: (a) Demographics; (b) Knowledge: comprised of 11 dichotomous (true/false) items to assess factual understanding of PrEP, (c) Attitudes and Perceptions: Each domain included six items measured on a 5-point Likert scale ranging from “Strongly Disagree” to “Strongly Agree.” The questionnaire can be found in Additional File 2.

### Data collection

The institutions and participants were primarily approached through faculty networks and professional connections of authors and the data collection team. This approach facilitated access to eligible participants from different medical and pharmaceutical teaching institutes while ensuring voluntary participation and informed consent. We approached a total of 10 institutes per city, out of which 5 institutes were offering medical programs and 5 were offering PharmD programs, but only 4 institutes per city consented to participate and provided the responses. Data was collected through quantitative surveys both in-person engagement of participants and via online surveys. Although the questionnaire was administered via Google Forms, most of participants completed the survey in person under the supervision of the research team and trained data collectors in the classroom settings. During these sessions participants were briefed about the study objectives and instructed to seek clarification for any unclear items while refraining from using externals sources such as internet. This approach ensured accurate understanding and reliable responses. The remaining data were obtained through online surveys circulated via social media platforms, including WhatsApp (WhatsApp LLC, Meta Platforms Inc., Menlo Park, California, United States) and Facebook (Meta Platforms Inc., Menlo Park, California, United States), accompanied by a brief introduction to the study, eligibility criteria, and an informed consent statement at the beginning of the survey forms.

### Data analysis

Data were initially exported from Google Forms into Microsoft Excel for cleaning, then imported into Statistical Package for Social Sciences (SPSS) version 27.0 (IBM Corporation, Armonk, New York, United States) for analysis. Descriptive statistics (frequencies and percentages) were used to summarize demographic characteristics. For knowledge, attitudes, and perception, the responses were scored for individual questions. In the knowledge section, each correct response was assigned a score of 1, while incorrect responses were scored as 0, yielding a minimum possible score of 0 and a maximum of 10. For attitudes and perceptions items, a 5-point Likert scale was used. Positively framed items were scored from 5 (Strongly Agree) to 1 (Strongly Disagree), and reverse scoring was applied for negatively worded items. For knowledge assessment, the total score was 10, scores less than or equal to 5 were categorized as low knowledge and scores more than 5 as high knowledge. The attitudes and perception domains each had a total possible score of 30 (range 6–30), scores less than or equal to 12 were considered negative attitudes/perception, whereas scores greater than 12 indicated positive attitude and perception. Aggregate scores for KAP were analyzed using means and standard deviations (SD). Due to non-normal distribution, the Mann-Whitney U test (for two-group comparisons) was used to examine associations between demographic variables and KAP scores, while Spearman’s rank correlation was applied to assess relationships among knowledge, attitudes, and perceptions scores.

### Ethics statement

Ethical approval for this study was obtained from the Institutional Review Board of Faisalabad Medical University, Faisalabad, Pakistan (Reference Number: 48.ERC/FMU/2024-25/69). All data collection procedures adhered to the ethical standards set forth by the committee. Informed consent was obtained prior to the survey; participants were informed about the objectives of the study and assured of confidentiality and voluntary participation. Respondents were told that they had the right to withdraw from the study at any point without any consequences, and the data would only be used for publication purposes. All study procedures utilized were in accordance with Principles of the Declaration of Helsinki, Good Clinical Practices, and within the applicable laws and regulations of research pertaining to human subjects in Pakistan.

## Results

A total of 450 eligible students were approached through faculty networks and connections of authors and data collection team. Following data cleaning and exclusion of incomplete or invalid responses, 359 complete responses were included in the final analysis resulting in an effective response rate of approximately 79.8%. Although multiple institutes from both the cities were approached, only a few institutes from each city consented to participate and provide complete responses.

### Baseline characteristics

Data from 359 participants were analyzed, the majority of whom (91.9%) had an age between 21 and 25 years, and the remaining 8.1% were aged 26 to 30 years. Gender wise, 58.5% participants were males and 41.5% were females. A notable fraction of participants (66.9%) was enrolled in the MBBS degree, while 33.1% were pharmacy students. According to the education year, 60.4% were fourth-year students, and 39.6% were studying in the final year. The participants from private institutions (54.6%) were more compared to participants from public institutions (45.4%). Regarding self-reported HIV PrEP awareness, 70.5% of participants were aware of PrEP, and the rest 29.5% reported unfamiliarity with HIV PrEP (Table 1).

**Table 1:** Demographic characteristics of participants (*n* = 359)

Variables	*n* (%)
Age	
21–25	330 (91.9%)
26–30	29 (8.1%)
Gender	
Male	210 (58.5%)
Female	149 (41.5%)
Degree	
MBBS	240 (66.9%)
Pharmacy	119 (33.1%)
Education Level	
Fourth Year	217 (60.4%)
Final Year	142 (39.6%)
Institute	
Public	163 (45.4%)
Private	196 (54.6%)
Awareness with HIV PrEP	
I am not aware	106 (29.5%)
I am aware	253 (70.5%)

### Knowledge-related characteristics of participants

The mean knowledge score came out to be 6.54 out of 10 (SD = 1.69) (Table 2), suggesting a moderate level of knowledge, reflecting high awareness about HIV PrEP but consistent misconception about its nature and testing requirements. In terms of individual questions, the majority have rightly identified that PrEP should be given to high-risk individuals only. Similarly, knowledge about particular candidate groups for HIV PrEP was good, with high recognition that sex workers (92.8%), PWID (89.1%), serodiscordant couples (85.2%), and transgender individuals (72.4%) are potential candidates for HIV PrEP. Almost Half of the participants (50.1%) mistakenly believed that PrEP was a vaccine. In addition, 56.3% believed that testing is not necessary before initiating PrEP, indicating misconceptions that could hamper safe and effective implementation of PrEP programs (Table 3).

**Table 2:** KAP scores among medical and pharmacy students regarding HIV PrEP

Variable	Mean (Standard Deviation)	Range
**Knowledge Score**	6.54 (1.69)	0–10
**Perception Score**	21.59 (3.72)	6–30
**Attitude Score**	23.35 (3.27)	6–30

**Table 3:** Responses to Knowledge-related questions regarding HIV PrEP

Sr	Question	True	False
1	Pre-exposure prophylaxis should be given to whole population	209 (58.2%)	150 (41.8%)
2	Pre-exposure prophylaxis should only be given to high-risk individuals i.e. people who are at risk of getting HIV infection.	281 (78.3%)	78 (21.7%)
3	PrEP is a vaccine.	180 (50.1%)	179 (49.9%)
4	HIV testing is not necessary prior to initiation of PrEP	202 (56.3%)	157 (43.7%)
5	PrEP can be used for prevention as well as treatment of HIV infection	124 (34.5%)	235 (65.5%)
6	PrEP provides immediate protection against HIV infection	133 (37.0%)	226 (63.0%)
7	Transgender individuals are potential candidates for PrEP.	260 (72.4%)	99 (27.6%)
8	Intravenous drug users are potential candidates for PrEP	320 (89.1%)	39 (10.9%)
9	Serodiscordant couples are potential candidates for PrEP	306 (85.2%)	53 (14.8%)
10	Sex workers are potential candidates for PrEP	333 (92.8%)	26 (7.2%)

### Perception-related characteristics of participants

The mean perception score was 21.59 (SD = 3.72) (Table 2), demonstrating an overall positive perception, and implies that most participants viewed PrEP as acceptable, safe, effective, and beneficial for HIV prevention. Almost half of the participants (51.8%) disapproved that increased focus on PrEP may divert funding and efforts from other HIV prevention strategies; however, a rather smaller fraction of 33.7% agreed with the view. Similarly, 65.7% of participants opposed the idea that healthcare providers lack of time and knowledge for HIV PrEP counselling. The notions of long-term use of PrEP leading to side effects, use of PrEP contributing to resistance against anti-retroviral drugs, ineffectiveness of PrEP due to lack of compliance, and PrEP application resulting in increased incidence of sexually transmitted diseases were rejected by 59.4%, 52.1%, 61.9% and 57.9% of participants, respectively (Table 4).

**Table 4:** Responses to Perception-related questions regarding HIV PrEP

Sr.	Questions	Strongly disagree	Disagree	Neutral	Agree	Strongly agree
1	PrEP may divert funding and efforts from other HIV prevention methods	60 (16.7%)	126 (35.1%)	87 (24.2%)	73 (20.3%)	13 (3.6%)
2	Healthcare providers may not be able to counsel individuals regarding PrEP due to lack of adequate knowledge and enough time.	68 (18.9%)	168 (46.8%)	72 (20.1%)	43 (12.0%)	8 (2.2%)
3	Long term use of PrEP in healthy people may lead to long term side effects forcing individuals to discontinue PrEP.	62 (17.3%)	151 (42.1%)	112 (31.2%)	27 (7.5%)	7 (1.9%)
4	Use of PrEP can lead to increased drug resistance against antiretroviral drugs.	65 (18.1%)	122 (34.0%)	122 (34.0%)	38 (10.6%)	12 (3.3%)
5	Lack of adherence to PrEP may render it ineffective	76 (21.2%)	146 (40.7%)	102 (28.4%)	29 (8.1%)	6 (1.7%)
6	Individuals receiving PrEP may abandon safe sex leading to increased STIs incidence.	73 (20.3%)	135 (37.6%)	98 (27.3%)	41 (11.4%)	12 (3.3%)

### Attitude-related characteristics of participants

For the attitudes segment, the mean score was 23.35 (SD = 3.27) (Table 2) out of 30, demonstrating a predominantly positive and supportive attitude towards the use, promotion and implementation of PrEP in Pakistan. In case individual question responses, 87.4% approved of promotion of PrEP in Pakistan, 84.7% supported free provision of PrEP at the Government’s expense and 82.7% agreed to the view that PrEP should be included in HIV prevention education. The ideas of PrEP users being perceived as HIV positive patients and PrEP being more expensive and less effective in real life were rejected by 73.6% and 54.6% participants. The question pertaining to PrEP advocacy for healthy individuals being unethical received mixed responses, with 35.5% opposing it, 30.9% endorsing it and the other 35.7% remaining neutral in this question (Table 5).

**Table 5:** Responses to Attitude-related questions regarding HIV PrEP

Sr.	Questions	Strongly Disagree	Disagree	Neutral	Agree	Strongly Agree
1	PrEP against HIV infection should be promoted and made widely available in Pakistan	5 (1.4%)	3 (0.8%)	37 (10.3%)	139 (38.7%)	175 (48.7%)
2	Provision of PrEP to high-risk individuals should be made by government of Pakistan free of cost.	4 (1.1%)	9 (2.5%)	42 (11.7%)	127 (35.4%)	177 (49.3%)
3	It will be unethical and not beneficial to recommend PrEP to healthy individuals.	51 (14.2%)	73 (20.3%)	128 (35.7%)	75 (20.9%)	32 (8.9%)
4	In my opinion, PrEP will be a more expensive and less effective preventive tool against HIV in real life.	68 (18.9%)	128 (35.7%)	109 (30.4%)	41 (11.4%)	13 (3.6%)
5	Individuals taking PrEP may be perceived as HIV positive patients by the community.	95 (26.5%)	169 (47.1%)	65 (18.1%)	25 (7.0%)	5 (1.4%)
6	PrEP must be an essential part of HIV prevention education.	5 (1.4%)	7 (1.9%)	50 (13.9%)	153 (42.6%)	144 (40.1%)

### Relation between Knowledge, perception, and attitude overall scores

Application of Spearman’s rank correlation reflected negative but statistically insignificant correlations between knowledge and perception scores (*r* =−0.100, *p* = 0.059). The relation between knowledge and attitude was also statistically insignificant (*r*=−0.102, *p* = 0.053). Nevertheless, the correlation between attitude and perception came out to be positive and statistically significant, reflecting students with a favorable attitude towards PrEP also perceived HIV PrEP in a positive sense (*r* = 0.555, *p* < 0.001) (Table 6).

**Table 6:** Spearman’s correlation between Knowledge, perceptions and attitudes regarding HIV PrEP among medical and pharmacy students

1 st Variable	2nd Variable	Spearman’s rho (Correlation Coefficient)	*p*-value
**Knowledge Scores**	**Perception Scores**	−0.100	0.059
**Knowledge Scores**	**Attitude Scores**	−0.102	0.053
**Perception Scores**	**Attitude Scores**	0.555^*^	< 0.001

* Correlation is significant at the 0.001 level (2-tailed)

### Relationship between demographics and overall scores

To assess relationship between demographics and overall knowledge, attitude, and perception scores, the Mann-Whitney U test was utilized. Gender wise, males secured higher mean rank scores in all three domains. This gender segregation, however, was statistically significant only in the case of the knowledge segment (Males = 192.41, Females = 162.52; *p* = 0.006). In the case of age groups, the test yielded no statistically significant association. Institute type seemed to have some effect on knowledge (*p* < 0.001) as mean rank scores were higher in public sector students (214.51) compared to private institute students (151.30) while the relation for attitude and perception was not statistically significant. Knowledge-wise medical students (200.97) scored notably higher (*p* = 0.001) than pharmacy students (137.71), while the scores for perception (Medical = 171.24, Pharmacy = 197.66, *p* = 0.023) and attitude (Medical = 164.26, Pharmacy = 211.75, *p* < 0.001) were statistically greater in Pharmacy students. In the case of year of education, no significant differences were obtained between fourth year and final-year students across all three segments. Awareness about PrEP did not significantly affect overall knowledge and perception scores; however, students aware of PrEP demonstrated slightly favorable attitudes regarding PrEP, although this result was statistically not significant (*p* = 0.086) (Table 7).

**Table 7:** Mann-Whitney U-Test results: demographics vs. Overall scores of Knowledge, attitude and perceptions

Variables (*n*)	Knowledge Scores		Perception Scores		Attitude Scores		
	Mean Rank	*p*-value	Mean Rank	*p*-value	Mean Rank		*p*-value
**Gender**							
Males (210)	192.41	0.006	182.54	0.581	183.33		0.468
Females (149)	162.51		176.43		175.30		
**Age**							
21–25 (330)	181.39	0.386	179.36	0.694	179.69		0.848
26–30 (29)	164.22		187.24		183.53		
**Institute Type**							
Public (163)	214.51	< 0.001	179.39	0.918	175.47		0.448
Private (196)	151.30		180.51		183.77		
**Degree**							
Medical (240)	200.97	< 0.001	171.24	0.023	164.26		< 0.001
Pharmacy (119)	137.71		197.66		211.75		
**Year of Study**							
Fourth (217)	184.82	0.269	176.57	0.437	172.02		0.070
Final (142)	172.64		185.24		`192.02		
**PrEP Awareness**							
Aware (253)	178.16	0.598	180.01	0.997	186.05		0.086
Not aware (106)	184.39		179.97		165.56		

## Discussion

The study provides one of the first insights into knowledge, attitude, and perceptions of PrEP among medical and pharmacy students in Pakistan. Overall, awareness of PrEP was encouragingly high, and participants demonstrated generally positive and supportive attitudes toward its use in HIV prevention. Nevertheless, significant knowledge gaps were observed regarding misconceptions about PrEP being a vaccine and the need for HIV testing prior to initiation. A clear difference was noted between disciplines, with medical students displaying stronger knowledge than pharmacy students, although both groups recognized key populations who would benefit from PrEP. Importantly, students expressed willingness to endorse PrEP promotion, free provision, and integration into HIV education, yet lingering concerns regarding stigma, cost, and ethical issues were also evident. These findings highlight both strengths and shortcomings in the preparedness of future healthcare providers and underscore the urgent need to strengthen HIV-related curricular content. By situating these results within Pakistan’s evolving PrEP landscape, this study contributes novel evidence to inform educational reform and implementation strategies aimed at optimizing PrEP uptake in resource-limited, high-stigma settings.

Differences between disciplines were notable. Medical students reported greater knowledge than pharmacy students; this pattern is consistent with several LMIC reports showing variable awareness and generally low knowledge of PrEP among health students; for example, some studies from Nigeria and Zambia reported awareness below 40% in certain student groups, and a pooled analysis across multiple African countries found high awareness but poor practical knowledge overall [32–35]. While awareness in our study was high, significant knowledge gaps were evident. This aligns with prior findings, which reported high awareness but limited knowledge about PrEP. More than half of the participants held misconceptions, such as confusing PrEP with a vaccine or misunderstanding pre-initiation testing, highlighting misinformation as a potential barrier to PrEP uptake within healthcare settings. Encouragingly, respondents demonstrated accurate recognition of potential PrEP candidates. This finding has also been observed in LMIC studies where students could identify high-risk groups despite limited technical knowledge [33, 34]. However, 52% of our study participants mistakenly believed that PrEP is a vaccine, illustrating critical educational gaps in public health training. The superior knowledge scores among medical students may, in part, be attributed to differences in curriculum. In Pakistan, the PharmD program lacks emphasis on clinical practice and public health training [36]. In contrast, medical students receive structured training in community medicine and clinical rotations, which is likely to contribute to their stronger knowledge of PrEP. Similar curriculum gaps have been documented across LMICs, where PrEP content is often absent or limited in undergraduate health professions training; targeted educational interventions in LMIC settings have produced measurable knowledge gains [37, 38]. It should also be noted that a comparatively smaller number of pharmacy students (*n* = 119) have participated in the study, which may have influenced the observed differences. Nonetheless, these findings highlight the need for a comprehensive revision of healthcare curricula, with a particular focus on enhancing public health components in pharmacy education, to better equip our future healthcare professionals in HIV prevention and care.

Interestingly, students pursuing pharmaceutical education in our study held a favorable perception of PrEP use. This positive attitude mirrors results from several LMIC surveys, where students expressed support for PrEP despite limited technical knowledge, although some LMIC studies report mixed views on resistance and safety [39, 40]. Unlike previous studies on pharmacy students [30] and providers [28], a majority in our sample rejected the misconception that long-term PrEP use promotes resistance to antiretrovirals (ARVs). This is an encouraging finding, as such misconceptions may otherwise deter support for PrEP. WHO guidelines and clinical trials have demonstrated that PrEP-related drug resistance is rare [3], a conclusion supported by a recent systematic review and meta-analysis [41]. Thus, the rejection of this myth represents a positive outcome of our study.

A concern surrounding PrEP is risk compensation, a notion that PrEP users may engage in riskier sexual behaviors, increasing their susceptibility to other sexually transmitted infections (STIs) [42]. Most of our respondents rejected the belief that PrEP leads to unsafe sexual practices. In some LMIC studies, including settings from Thailand and other parts of Asia, a substantial proportion of providers and students reported concern that PrEP could encourage riskier behavior, which can create hesitancy despite recognition of PrEP’s benefits [43, 44]. Though mixed evidence exists [45, 46], CDC and WHO recommended combining PrEP with behavioral risk-reduction strategies [3, 5]. Therefore, concerns about risk compensation should not hinder PrEP provision to key populations.

Most participants did not perceive PrEP as diverting resources further away from other prevention strategies, a perspective that aligns with findings among Ethiopian healthcare professionals [47]. However, resource concerns are common across LMICs; limited funding, cost barriers for patients, and constrained clinic capacity frequently surface as barriers for PrEP scale-up [48]. While Pakistan was previously a primary recipient of Global Fund (GF) support for HIV, tuberculosis, and malaria, the recent reduction in U.S. contributions to UNAIDS [49] has strained available resources. Although PrEP is a powerful prevention tool, resource allocation must be balanced to avoid neglecting broader prevention strategies. Disparities in access, particularly among ethnic minorities and marginalized communities, could intensify if prevention efforts are disproportionately focused on PrEP [50]. However, there is no documented evidence that PrEP funding has led to reductions in other HIV prevention services, lending some support to participants’ positive perceptions.

Overall, students expressed supportive attitudes toward PrEP, with pharmacy students in particular demonstrating slightly more favorable dispositions than their medical counterparts. A significant number of respondents advocated for PrEP promotion. LMIC experience shows low uptake and narrow targeting in practice; many national programs prioritize men who have sex with men, while other at-risk groups remain underserved, which helps explain why national program launch has not yet produced high uptake in Pakistan [39, 44, 51]. Promotion of both traditional oral and emerging long-acting PrEP formulations is crucial for maximizing impact. Although CDC and WHO recommend PrEP for all key populations [3, 5], implementation has predominantly targeted MSM, often excluding other at-risk groups [52]. Our findings indicate a positive outlook among students regarding broader advocacy for PrEP.

A considerable proportion of students believed that PrEP users may be perceived as HIV positive. Similar misconceptions and stigma have been reported across LMIC studies, where PrEP users are frequently assumed to be HIV positive, and this reduces willingness to seek PrEP [43, 44]. Although this misconception has been rejected by other studies [47, 53], its persistence highlights the deep-rooted influence of societal stigma. PrEP uptake and adherence are influenced by internal factors (e.g., homophobia, individual beliefs) and external factors (e.g., shaming, community stigma) [54]. Previous research confirms that PrEP users are often mistakenly seen as HIV-positive [55]. Such stigma poses a significant barrier to both PrEP access and retention in care [56]. To address this, a non-stigmatizing and supportive environment must be fostered [57].

A notable portion believed that PrEP is expensive but not cost-effective. However, studies show that PrEP can be highly cost-effective, particularly when coverage is expanded and services are targeted to key populations [50, 58]. Therefore, the perception of PrEP as a financially burdensome intervention is not supported by evidence.

Encouragingly, students expressed strong interest in including PrEP within HIV education curricula. Similar interest and positive responses to education have been observed in LMICs; for example, an online PrEP training in Malaysia produced a significant mean increase in knowledge scores [38]. In Pakistan, PrEP education is absent from both medical [59] and pharmacy curricula [60]. Given these gaps, integrating real-world HIV training during clinical education phases is essential to prepare future professionals. Thus, participants’ eagerness to learn about PrEP is a promising outcome of this study.

Students also voiced concerns about the ethical implications of promoting PrEP among healthy individuals. However, in Pakistan’s concentrated epidemic, individuals such as PWID, sex-workers, MSM, transgender sex workers, and recipients of infected blood appear healthy but are at high risk [61, 62]. CDC and WHO guidelines support PrEP use in such at-risk populations. Therefore, educational interventions are essential to counter stigma and misinformation.

Our findings have several implications for improving medical and pharmacy education in Pakistan. While medical students showed better knowledge of PrEP, misconceptions, particularly regarding cost-effectiveness and benefits, remain prevalent. These observations highlight the need for curricular reform focused on evidence-based HIV education. Case-based learning modules, focused on HIV risk assessment, PrEP initiation, and management, are available from reputable sources, including Fenway Institute [63] and National HIV Curriculum (NHIVC), University of Washington [64]. These resources can be tailored to medical and pharmacy education in Pakistan. Evidence from LMICs shows that blended learning and short online modules are feasible and effective at improving PrEP knowledge and prescribing intentions; these approaches could be adapted for Pakistan’s undergraduate curricula. Since the country adheres to WHO guidelines developed for LMICs, national HIV education programs must be established for healthcare training and awareness. Furthermore, with the approval of long-acting injectable PrEP, such as cabotegravir [65] and inclusion of biannual Lenacapavir injections in the WHO guidelines [66], educators must stay updated on advancements in HIV prevention. Based on our findings, a summary of key recommendations for improving PrEP-related education, awareness, and implementation is provided below (Table 8).

**Table 8:** Summarization of key recommendations for HIV PrEP education in Pakistan

Domain	Recommendation
**Curriculum Development**	Integrate comprehensive HIV educational modules into medical and pharmacy curricula, focusing on prevention, risk-reduction, and treatment.
**Pharmacy Education**	Revise the PharmD curriculum to include pharmacy practice, public health, and HIV related content.
**Faculty Development**	Conduct training workshops for educators on modern HIV prevention strategies, including PrEP and long-acting injectable options
**Stigma Reduction**	Introducing sensitization sessions on stigma, ethics, and community perceptions regarding PrEP use
**Public Health Advocacy**	Promote PrEP among all key populations through national awareness campaigns.
**Evidence-Based Policy Making**	Encourage Policymakers to allocate balanced resources for PrEP without compromising other HIV prevention services
**Case-based and Clinical Training**	Implement case-based learning and clinical simulations in HIV risk assessment and PrEP provision,
**Access to Learning Resources**	Recommend the adoption of online modules such as those from the Fenway Institute and National HIV Curriculum by the University of Washington.
**National HIV Education Program**	Develop a national standardized HIV education initiative aligned with WHO guidelines

### Strengths and limitations

Our study has several strengths that enhance its relevance and contribute to existing literature. Notably, it is the first study in Pakistan to assess the knowledge, attitude, and perceptions of HIV PrEP among medical and pharmacy students, who populations regarded as the future healthcare force. Including students in the terminal phase of their degree programs ensures the data reflects the perspectives of the individuals soon to enter healthcare practice. Given Pakistan’s increasing burden of HIV infections [61]. This study addresses a critical gap in how future professionals perceive and are prepared to engage with PrEP.

Although the study has several strengths, some limitations must be acknowledged. Our study does not involve students among other healthcare degrees, like nursing students. Nursing professionals play a significant role in comprehensive healthcare delivery, collaboratively with medical and pharmacy professionals [67]. A similar study in the United States involved nursing students in their study [10]. Secondly, our study utilizes a cross-sectional methodology that restricts any causal inferences and leads to self-reporting data, which can generate social desirability. Additionally, the sample may not fully capture the diversity of all healthcare training institutes across Pakistan. Finally, there were fewer pharmacy students who participated in the study as compared to medical students, which may have influenced the comparative findings and contributed to the lower knowledge levels and higher perceptions and attitudes scores observed among pharmacy students. Despite these limitations, the study offers valuable insights into the existing gaps in knowledge, perceptions, and attitudes towards HIV PrEP among medical and pharmacy students in Pakistan, underscoring the need for targeted educational interventions. Future studies should prioritize nationwide, multisite studies or the use of a mixed-methods approach involving quantitative and qualitative studies to gain a better understanding of the dilemma surrounding HIV education among healthcare undergraduates.

## Conclusion

In light of Pakistan’s persistent HIV burden and the underutilization of PrEP, our study offers critical insights into the factors influencing PrEP uptake among future healthcare providers. Although participants demonstrated a moderate level of knowledge, significant gaps remain, particularly among pharmacy students. Encouragingly, both medical and pharmacy students exhibited overwhelmingly positive attitudes and perceptions towards PrEP, underscoring their readiness to champion HIV prevention. The higher knowledge scores observed in medical students highlight an urgent need to enrich the PharmD curriculum with targeted, public health-oriented HIV education. Collectively, these findings demand a multifaceted response: comprehensive curriculum reform, immersive case-based learning, government-led HIV awareness campaigns, and establishment of competency-based certification programs. By implementing these strategies, we can empower healthcare students to drive PrEP adoption and, ultimately, curb the trajectory of the HIV epidemic in Pakistan.

## Supplementary Information

Supplementary Material 1

Supplementary Material 2

## Data Availability

Data will be available upon reasonable request by contacting the corresponding author.

## Abbreviations

AIDS: Acquired Immunodeficiency Syndrome
ART: Antiretroviral Therapy
ARVs: Antiretrovirals
CAB-LA: Cabotegravir Long-Acting Injectable
CDC: Centers for Disease Control and Prevention
COVID-19: Coronavirus Disease 2019
DVR: Dapavirine Vaginal Ring
HIV: Human Immunodeficiency Virus
GF: Global Fund
KAP: Knowledge, Attitudes and Perceptions
KPs: Key Populations
LMICs: Low- or Middle-Income Countries
MBBS: Bachelor of Medicine and Bachelor of Surgery
MSM: Men who have Sex with Men
NACP: National AIDS Control Program, Pakistan
PharmD: Doctor of Pharmacy
PrEP: pre-exposure Prophylaxis
PWH: People with HIV
PWID: People Who Inject Drugs
SD: Standard Deviation
STROBE: Strengthening the Reporting of Observational Studies in Epidemiology
UNAIDS: Joint United Nations Programme on HIV/AIDS
U.S: United States
WHO: World Health Organization
