# Supplementary material for: Knowledge, attitutes and perceptions of medical and pharmacy students about HIV pre-exposure prophylaxis: a cross-sectional survey from Pakistan
Source: BMC Med Educ. 2025 Nov 29;26:4. doi: 10.1186/s12909-025-08338-6 (PMC12771764; doi:10.1186/s12909-025-08338-6)
Supplement: Supplementary file 2 — Supplementary Material 2 [file 12909_2025_8338_MOESM2_ESM.docx]

**Supplementary material 2: Study Questionnaire**

**Informed Consent**

This is a questionnaire-based study that aims to assess knowledge, attitude and perception of medical and pharmacy students about HIV pre-exposure prophylaxis in Pakistan. You are being asked to take part in this research study. Your participation is this study is purely voluntary. You have the right to decline or discontinue your participation at any time. Nevertheless, the data obtained from this research will be kept confidential and only used for research purpose.

**Signature:** ………………………… **Date:** …………………………

**Demographics**

**1. Gender: 1)** Male **2)** Female

**2. Age (in years): 1)** 15-20 **2)** 21-25 **3)** 26-30 **4)** > 30

**4) Degree:**  **1)** MBBS **2)** Pharmacy

**3. Year of Education: 1)** Second last **2)** Final

**5. Institute: 1)** Government **2)** Private

**6. Institute name: ……………………………………….**

**7. I am well aware about HIV Pre-exposure prophylaxis (PrEP): 1)** Government **2)** Private

**Awareness assessing questions.**

| Sr. | Question | **Yes** | **No** |
| --- | --- | --- | --- |
| **1** | Pre-exposure prophylaxis should be given to whole population. |  |  |
| **2** | Pre-exposure prophylaxis should only be given to high-risk individuals i.e. people who are at risk of getting HIV infection. |  |  |
| **3** | PrEP is a vaccine. |  |  |
| **4** | HIV testing is not necessary prior to initiation of PrEP. |  |  |
| **5** | PrEP can be used for prevention as well as treatment of HIV infection. |  |  |
| **6** | PrEP provides immediate protection against HIV infection. |  |  |
| **7** | Transgenders are potential candidates for PrEP. |  |  |
| **8** | Intravenous drug users are potential candidates for PrEP. |  |  |
| **9** | Serodiscordant couples are potential candidates for PrEP. |  |  |
| **10** | Sexworkers are potential candidates for PrEP. |  |  |

**Perception assessment questions:**

Select the most appropriate option according to following sequence:

| Sr. | Questions | Strongly  Agree | Agree | Neutral | Disagree | Strongly disagree |
| --- | --- | --- | --- | --- | --- | --- |
| **12** | PrEP may divert funding and efforts from other HIV prevention methods. |  |  |  |  |  |
| **13** | Healthcare providers may not be able to counsel individuals regarding PrEP due to lack of adequate knowledge and enough time. |  |  |  |  |  |
| **14** | Long term use of PrEP in healthy people may lead to long term side effects forcing individuals discontinue PrEP. |  |  |  |  |  |
| **15** | Use of PrEP can lead to increased drug resistance against antiretroviral drugs |  |  |  |  |  |
| **16** | Lack of adherence to PrEP may render it ineffective. |  |  |  |  |  |
| **17** | Individuals receiving PrEP may abandon safe sex leading to increased STIs incidence. |  |  |  |  |  |

**Attitude assessment questions:**

| Sr. | Questions | Strongly  Agree | Agree | Neutral | Disagree | Strongly disagree |
| --- | --- | --- | --- | --- | --- | --- |
| **18** | PrEP against HIV infection should be promoted and made widely available in Pakistan. |  |  |  |  |  |
| **19** | Provision of PrEP to high-risk individuals should be made by government of Pakistan free of cost. |  |  |  |  |  |
| **20** | Advocating PrEP for healthy individuals is unethical. |  |  |  |  |  |
| **21** | In my opinion, PrEP will be a more expensive and less effective preventive tool against HIV in real life. |  |  |  |  |  |
| **22** | Individuals taking PrEP may be perceived as HIV positive patients by the community. |  |  |  |  |  |
| **23** | PrEP must be an essential part of HIV prevention education. |  |  |  |  |  |
